# Promoting Mental Health of Nurses During the Coronavirus Pandemic: Will the Rapid Deployment of Nurses' Training Programs During COVID-19 Improve Self-Efficacy and Reduce Anxiety?

**DOI:** 10.7759/cureus.15213

**Published:** 2021-05-24

**Authors:** Sanjana Dharra, Rajesh Kumar

**Affiliations:** 1 Department of Nursing, All India Institute of Medical Sciences, Rishikesh, Rishikesh, IND

**Keywords:** self-efficacy, nurses, anxiety, covid-19, training

## Abstract

Introduction

The nursing profession is extremely demanding and stressful. Nurses have been facing a tremendous amount of fear and anxiety during the ongoing coronavirus disease 2019 (COVID-19) pandemic. General self-efficacy is considered one of the most influential factors that can modify the impact of anxiety on nurses' mental health. In this study, we aimed to determine the predictors of self-efficacy and anxiety among nurses during the current COVID-19 pandemic.

Methods

We conducted a cross-sectional survey involving 368 nurses working in a tertiary care hospital. The General Self-Efficacy scale (GSE) and Generalized Anxiety Disorder Scale-7 (GAD-7) were used for assessing self-efficacy and anxiety. The chi-square test followed by bivariate and multivariable regression analyses was performed to identify the determinants of self-efficacy and anxiety.

Results

The mean age of the nurses was 28.91 years (SD=3.68), with a mean working experience in the nursing field of 5.32 years (SD=2.48). The mean self-efficacy and anxiety scores were 32.19 (SD=4.53) and 3.82 (SD=2.87), respectively. Multivariable analysis showed that higher age (>30 years, p=.003), professional education acquired through a diploma course instead of a graduate degree (p<.001), and lack of training on handling COVID-19 patients (p=.003) were significant determinants of higher anxiety among nurses. Similarly, higher economic status (p=.001), sufficient COVID-19 training (p=.049), having family members tested positive for COVID-19 (p=.012), professional experience (≤5 years, p<0.001), and quarantine period (≤14 days, p=.002) were revealed to be independent determinants of a higher sense of self-efficacy among nurses during the COVID-19 pandemic.

Conclusion

Receiving adequate levels of training on COVID-19 plays a vital role in improving self-efficacy and reducing anxiety among nurses during the ongoing pandemic. Managing anxiety, increasing self-efficacy, and ensuring more exposure to COVID-19-related training may improve nurses' mental health and prepare them for fighting pandemics in a much better and more efficient manner.

## Introduction

The ongoing pandemic caused by severe acute respiratory syndrome coronavirus 2 (SARS-CoV-2) has put the efficiency of healthcare systems around the world to the test [1]. By March 28, 2021, there were 27 million confirmed cases of coronavirus disease 2019 (COVID-19) in 219 countries, and 2.78 million lives had been lost [2]. The sudden outbreak of this pandemic has overwhelmed and shaken the healthcare system to its core and severely jeopardized the routine management and provision of healthcare services and drastically endangered the health and wellbeing of frontline healthcare workers (HCWs) such as nurses [3].

Nurses form the backbone of the healthcare system and constitute a significant part of the trained workforce; they play a pivotal role in the provision of public healthcare [4]. A study has revealed that the Indian healthcare system is facing a severe shortage of trained nursing workforce [5]. Crucially, this shortage has been further intensified by a large number of nurses contracting COVID-19 during this pandemic and being forced quarantine as a result [5,6]. Studies from India [7] and Wuhan, China [8] have revealed that HCWs are often experiencing a great deal of anxiety, stress, and depression due to the current pandemic. HCWs, especially nurses, who are directly involved in the diagnosis, treatment, and care of patients with COVID-19 have been forced to work under varying and punishing time schedules and often been shifted to other departments to meet the crippling staff shortages [9]. From time to time, nurses have faced a shortage of personal protective equipment and have been forced to work without appropriate safety measures in place [10]. The lack of appropriate personal protective measures, fear of contracting the infection by self and family members, and the pressure due to higher workload have made them significantly vulnerable to an increased risk of psychological distress and other mental health issues [11].

In China, more than three-fourth of HCWs have reported experiencing psychological issues during this pandemic, including anxiety, insomnia, and depression [12]. Also, social distancing and isolation measures have fueled the symptoms of poor psychological health and other mental health issues [13]. It is reasonable to assume that strengthening the protective measures and procedures, such as providing encouragement to use positive coping styles and implementing measures to improve self-efficacy and resilience could protect the nurses from undergoing severe mental distress and improve their general mental wellbeing to a large extent [14].

Self-efficacy refers to an individual's ability to adjust to various ups and downs of an environment and the capacity to adapt to the challenges or new changes [9]. It will promote a greater sense of self-control in individuals, helping them to build up resilience and other positive psychosocial traits [15]. Previous research has reported a direct link between self-efficacy and mental health as well as an individual's overall mental well-being [1,16,17].

## Materials and methods

A descriptive cross-sectional study design was employed to assess the nurses' anxiety and self-efficacy levels at the All India Institutes of Medical Sciences (AIIMS), Rishikesh, Uttarakhand. Nurses working as direct care providers and performed duty during the COVID-19 outbreak were included by using a convenience consecutive sampling approach. Nurses who were under quarantine or those who had just reported back to work after quarantine were excluded from the study. The survey questionnaire was distributed to 500 nurses, and 400 nurses responded to them (80.0%). Finally, 368 questionnaires were deemed worthy of inclusion for the study after excluding incomplete or partially filled questionnaires. 

Ethical consideration

The Institutional Ethics Committee at AIIMS approved the study (AIIMS/IEC/20/799). Obtaining consent was made mandatory to participate in the survey. However, researchers avoided collecting any personal information of the participants to ensure confidentiality and protect the participants' privacy. 

Data collection tools

Sociodemographic Profile Sheet

This was designed as a tool to collect data comprising details of age, gender, education, professional qualification, living status, training on COVID-19, nursing experience, working area, family type, socioeconomic status, residential area, contacts with laboratory-confirmed COVID-19 cases, whether tested positive for COVID-19, quarantine history and days, history of family members being tested positive, and whether there were any medical professionals in the family. A group of experts in the fields of nursing, family medicine, and internal medicine was asked to validate the profiles gathered. The inclusion of items was based on experts reaching 100% agreement and consensus regarding their validity and relevance.

The Generalized Anxiety Disorder Scale-7 (GAD-7) 

The GAD-7 is a seven-item scale developed by Spitzer et al. (2006) [18]. In this, participants rate each item using a 4-point Likert scale, from 0 (not at all) to 3 (nearly every day), with a total score range of 0-21. When screening for anxiety disorders, a recommended cut-off score that is equal to or more than 7 is generally used [12]. This scale is a well-validated and efficient tool for screening anxiety disorders and has been widely used previously among similar populations [12].

The General Self-Efficacy (GSE) Scale 

The GSE scale is a 10-item scale developed to measure emotion, optimism, and work satisfaction [19]. The test-retest reliability value ranges from 0.69 to 0.80 [20]. A cut-off score of less than 29 is considered to indicate low self-efficacy, and that of more than 30 is considered to reflect high self-efficacy in the study [9].

Sample Size Analysis

A sample size calculation was performed by assuming a 29% prevalence of anxiety among nurses [21]. The sample size came out to be 316 and, adjusting for 10% attrition, it was finalized to be 348. A precision of 5% and 71% outcome factor in the population were presumed. However, the sample size achieved in the study was 368.

Statistical analysis

A datasheet was prepared on Microsoft Excel and analyzed using IBM SPSS Statistics for Windows, Version 23.0 (IBM, Armonk, NY) [22]. Descriptive statistics, frequencies, and percentages were used to present the characteristics of the participants. The chi-square and Fisher's exact tests were used as and when appropriate to find the factors associated with self-efficacy and anxiety among nurses. Bivariate and multivariable regression analyses were applied to quantify the strength of the association between sociodemographic characteristics of nurses and levels of self-efficacy and anxiety. The level of significance was set at a p-value of <0.05 (two-sided).

## Results

Table 1 depicts the personal and professional characteristics of the nurses. The mean age of the participants was 28.91 (±3.68) years. More than half of the participants (74.5%) were aged 30 years or less and 58.6% were females. More than half of the participants (57.0%) had acquired their qualification in nursing via a graduate degree and 27.3% were involved with direct care of COVID-19 patients. Of note, 61% of the participants had attended training programs on COVID-19 (virtual or in-person training on critical issues, i.e., airway, ventilation, ventilator, use of personal protective equipment, social-distancing norms, etc.), and 58.6% had come into contact with a laboratory-confirmed COVID-19 case; 38.5% of the participants had tested positive for COVID-19 themselves and had quarantined (72.5%) with a mean duration of 13.81 (±6.14) days. And 15.5% of the nurses had family members who had tested positive for COVID-19. Less than half (47.9%) of the nurses had family members in the medical profession; of them, 26% were in the nursing profession. The mean experience of the participants was 5.32 (SD=2.48) years. The mean self-efficacy score was 32.19 (SD=4.53), and the mean anxiety score was 3.82 (SD=2.87). The majority of the nurses (73.6%) displayed high self-efficacy, followed by 26.4% showing low self-efficacy; 9.2% of the nurses expressed symptoms of anxiety. 

As reported in Table 1, there was a statistically significant association between self-efficacy and age (p=0.014), marital status (p=0.009), working area in the hospital (p=0.019), family types (p=0.013), socioeconomic status (p=0.015), residential area (p=0.025), COVID-19 training (p=0.001), family members being tested positive for COVID-19 (p=0.007), having family members in the medical profession (p=0.001), professional experience (p<0.001), and the number of quarantine days (p=0.009) among the nurses during the pandemic.

Furthermore, anxiety among nurses showed a statistically significant association with age (p<0.001), gender (p=0.001), professional education (p<0.001), working area (p=0.003), COVID-19 training (p<0.001), contact with a lab-confirmed COVID-19 case (p=0.020), quarantine history (p=0.001), family members in the medical profession (p=0.046), and years of experience (p=0.005) (Table 1).

**Table 1:** Association of sociodemographic characteristics with self-efficacy and anxiety among nurses (n=368) *Significant p-value at <0.05; ^ᶲ^nurses with MSc nursing qualification were excluded from analysis due to negligible numbers; ^ǂ^as per Kuppuswamy SES scale; ^$^lab-confirmed COVID-19 case; ^Fisher's exact test; ^§^based on GSE scale; ^¶^based on GAD scale COVID-19: coronavirus disease 2019; SES: socioeconomic status; GSE: General Self-Efficacy; GAD: Generalized Anxiety Disorder

Variables	Categories	Total, n (%)	Self-efficacy^§^		Anxiety^¶^	
			Low, n (%)	P-value	Yes, n (%)	P-value
Age (years; mean: 28.91 ±3.68)	≤30	275 (74.7)	80 (82.5)	0.014*	16 (47.1)	<0.001*
	>30	93 (25.3)	17 (17.5)		18 (52.8)	
Gender	Male	149 (39.8)	35 (36.1)	0.303	5 (14.7)	0.001*
	Female	219 (58.6)	62 (63.9)		29 (85.3)	
Marital status	Married	233 (62.3)	72 (74.2)	0.009*	25 (73.5)	0.195
	Unmarried	135 (36.1)	25 (25.8)		09 (26.5)	
Professional education (n=332)ᶲ	Diploma in nursing	123 (32.9)	43 (44.3)	0.078	24 (82.8)	<0.001
	Graduate degree	209 (55.9)	53 (55.7)		05 (17.2)	
Working area	COVID-19	02 (27.3)	18 (18.6)	0.019*	02 (5.9)	0.003*^
	Non-COVID-19	266 (71.1)	79 (81.4)		32 (94.1)	
Family type	Nuclear	189 (50.4)	37 (38.1)	0.013*	18 (52.9)	0.852
	Joint	179 (47.9)	60 (61.9)		16 (47.0)	
Socioeconomic status^ǂ^	Upper middle class	306 (83.2)	73 (75.3)	0.015*	26 (76.5)	0.275
	Lower middle class	62 (16.8)	24 (24.7)		08 (23.5)	
Residential area	Within hospital taluk	291 (77.8)	69 (71.1)	0.025*	23 (67.6)	0.085
	Outside of taluk premises	77 (20.6)	28 (28.9)		11 (32.4)	
COVID-19 training	Yes	228 (61.0)	46 (47.4)	0.001*	08 (23.5)	<0.001*
	No	140 (37.4)	57 (52.6)		26 (76.5)	
Contact with COVID-19-positive patients^$^	Yes	219 (58.6)	65 (67)	0.091	14 (41.2)	0.020*
	No	149 (39.8)	32 (33)		20 (58.8)	
Tested COVID-19-positive	No	144 (38.5)	38 (39.2)	0.992	14 (41.2)	0.797
	Yes	224 (59.9)	59 (60.8)		20 (58.8)	
Quarantine history	No	271 (72.5)	72 (74.2)	0.879	17 (50.0)	0.001*
	Yes	97 (25.9)	25 (25.8)		17 (50.0)	
Family tested COVID-19-positive	Yes	58 (15.5)	07 (7.21)	0.007*	04 (11.8)	0.502^
	No	310 (82.9)	90 (92.8)		30 (88.2)	
Medical professionals in the family	Yes	179 (47.9)	33 (34.0)	0.001*	11 (33.4)	0.046*
	No	189 (50.5)	64 (66.0)		23 (67.6)	
Professional experience (years; mean: 5.32 ±2.48)	≤5	224 (60.9)	42 (43.3)	<0.001	13 (38.2)	0.005*
	>6	144 (39.1)	55 (56.7)		12 (61.8)	
Quarantine days (n=271; mean: 13.81 ±6.14)	≤14	220 (59.8)	51 (70.8)	0.009*	15 (88.2)	0.748^
	>15	51 (13.9)	21 (39.2)		02 (11.8)	

Additionally, bivariate logistic regression was applied to quantify the strength of association between sociodemographic characteristics of nurses and their self-efficacy status. All the variables that showed significant association were entered into a bivariate regression model.

The findings revealed that self-efficacy levels were lower in younger nurses (<30 years) compared to older nurses (OR: 0.545, 95% CI: 0.303-0.980, p=0.043). Married nurses showed low self-efficacy levels compared to unmarried nurses (OR: 0.508, 95% CI: 0303-0.851, p=0.010). Nurses who belonged to the nuclear family system and those with upper-middle-class economic status reported higher status of self-efficacy as compared to their counterparts (OR: 1.812, 95% CI: 1.128-2.910, p=0.014; OR: 2.016, 95% CI: 1.135-3.581, p=0.017, respectively). Nurses working in the COVID-19 area and those living within taluk premises of the hospital showed higher self-efficacy compared to nursing working in non-COVID-19 areas and those staying outside of taluk premises of the hospital (OR: 1.971, 95% CI: 1.112-3.497, p=0.020; OR: 1.839, 95% CI: 1.074-3.146, p=0.026, respectively). Nurses who had not attended training on COVID-19 reported lower self-efficacy compared to nurses who had attended training on COVID-19 (OR: 0.441, 95% CI: 0.275-0.707, p=.001). Nurses whose family members had tested positive for COVID-19 reported higher self-efficacy than the nurses whose family members had not had an infection (OR: 0.336, 95% CI: 0.146-0.767, p=0.010). Nurses with professional experience of less than or equal to five years and those who had stayed in quarantine for less than 14 days reported levels of self-efficacy two times higher compared to their counterparts (OR: 2.678, 95% CI: 1.665-4.306, p<0.001; OR: 2.320, 95% CI: 1.224-4.397, p=0.010, respectively) (Table 2).

**Table 2:** Bivariate regression analysis to find the factors associated with self-efficacy levels among nurses (N=368) *Significant p-value at <0.05; ^#^as per Kuppuswamy SES scale COVID-19: coronavirus disease 2019; SES: socioeconomic status; OR: odds ratio; CI: confidence interval

Variables	Categories	OR (95% CI)	P-value
Age (years)	≤30	0.545 (0.303-0.980)	0.043*
	>30	Reference (1)	
Marital status	Married	0.508 (0.303-0.851)	0.010*
	Unmarried	Reference (1)	
Working area	COVID-19	1.971 (1.112-3.497)	0.020*
	Non-COVID-19	Reference (1)	
Family type	Nuclear	1.812 (1.128-2.910)	0.014*
	Joint	Reference (1)	
Socioeconomic status^#^	Upper middle class	2.016 (1.135-3.581)	0.017*
	Lower middle class	Reference (1)	
Living area	Within taluk premises	1.839 (1.074-3.146)	0.026*
	Not in taluk premises	Reference (1)	
COVID-19 training	No	0.441 (0.275-0.707)	0.001*
	Yes	Reference (1)	
Family members tested COVID-19-positive	No	0.336 (0.146-0.767)	0.010
	Yes	Reference (1)	
Professional experience (years)	≤5	2.678 (1.665-4.306)	<0.001
	>6	Reference (1)	
Quarantine days	≤14	2.320 (1.224-4.397)	0.010*
	>15	Reference (1)	

Multivariable logistic regression analysis was employed to see the combined effect of nurses' sociodemographic characteristics on self-efficacy during the pandemic. The findings revealed that nurses staying within the hospital's taluk premises had levels of self-efficacy three times higher than the nurses staying outside the taluk of the hospital (OR: 3.811, 95% CI: 1.512-9.604, p=0.005). Nurses who belonged to higher socioeconomic status had levels of self-efficacy six times higher compared to nurses coming from lower-middle-class socioeconomic strata (OR: 5.959, 95% CI: 2.025-17.529, p=0.001). Also, nurses who had not attended training on COVID-19 had lower self-efficacy than nurses who had attended training on the different aspects of care and prevention of COVID-19 (OR: 0.464, 95% CI: 0.216-0.996, p=0.049). Nurses with professional experience of less than or equal to five years and those who had stayed in quarantine for less than 14 days after getting tested positive reported levels of self-efficacy four times higher than their counterparts (OR: 4.599, 95% CI: 2.093-10.105, p<0.001; OR: 4.179, 95% CI: 1.622-10.509, p=0.002, respectively). Nurses whose family members had tested positive for COVID-19 reported higher self-efficacy compared to those whose family members had not contracted the disease (OR: 0.226, 95% CI: 0.071-0.721: p=0.012) (Table 3).

**Table 3:** Multivariable regression analysis to find the factors associated with self-efficacy among nurses (n=368) *Significant p-value at <0.05; ^#^as per Kuppuswamy SES scale COVID-19: coronavirus disease 2019; SES: socioeconomic status; OR: odds ratio; CI: confidence interval

Variables	Categories	OR (95%: CI)	P-value
Age (years)	≤30	0.506 (0.198-1.294)	0.155
	>30	Reference (1)	
Marital status	Married	0.595 (0.246-1.435)	0.248
	Unmarried	Reference (1)	
Working area	COVID-19	21.49 (0.966-4.779)	0.061
	Non-COVID-19	Reference (1)	
Family type	Nuclear	0.744 (0.366-1.510)	0.413
	Joint	Reference (1)	
Socioeconomic status^#^	Upper middle class	5.959 (2.025-17.529)	0.001*
	Lower middle class	Reference (1)	
Living area	Within taluk premises	3.811 (1.512-9.604)	0.005*
	Not in taluk premises	Reference (1)	
COVID-19 training	No	0.464 (0.216-0.996)	0.049*
	Yes	Reference (1)	
Family members tested COVID-19-positive	No	0.226 (0.071-0.721)	0.012*
	Yes	Reference (1)	
Professional experience (years)	≤5	4.599 (2.093-10.105)	<0.001
	>6	Reference (1)	
Quarantine days	≤14	4.179 (1.662-10.509)	0.002*
	>15	Reference (1)	

Further, bivariate logistic regression was applied to quantify the strength of the association of factors affecting nurses' anxiety status. The findings revealed that younger nurses (<30 years) and males reported lower levels of anxiety compared to their counterparts (OR: 0.257, 95% CI: 0.125-0.529, p<0.001; OR: 0.227, 95% CI: 0.086-0.062, p=0.003, respectively). Likewise, nurses who had done their diploma education in nursing reported levels of anxiety nine times higher than graduate nurses (OR: 9.891, 95% CI: 3.664-26.698, p<0.001). Nurses who had not undergone training on COVID-19 had levels of anxiety six times higher compared to those who had received training (OR: 6.272, 95% CI: 2.751-14.298, p<0.001). Nurses who had not had contact with a laboratory-confirmed COVID-19 case and those who had stayed in quarantine for less than 14 days after testing COVID-19 positive reported higher levels of anxiety than the nurses who had come in contact with a laboratory-confirmed case and those who had spent time in quarantine for more than 14 days (OR: 2.299, 95% CI: 1.222-4.713, p=0.023; OR, 3.175, 95% CI: 1.549-6.508, p=0.002, respectively). Nurses with experience of less than or equal to five years and those working in the COVID-19 area felt less anxious compared to nurses with higher experience and those working in the non-COVID area (OR: 0.361, 95% CI: 0.175-0.746, p=0.006; OR: 0.146, 95% CI: 0.034-0.622, p=0.009, respectively) (Table 4).

**Table 4:** Bivariate regression analysis to find the factors associated with anxiety levels among nurses (n=368) *Significant p-value at <0.05; ^#^as per Kuppuswamy SES scale; ^$^lab-confirmed COVID-19 cases COVID-19: coronavirus disease 2019; SES: socioeconomic status; OR: odds ratio; CI: confidence interval

Variables	Categories	OR (95% CI)	P-value
Age (years)	≤30	0.257 (0.125-0.529)	<0.001
	>30	Reference (1)	
Gender	Male	0.227 (0.086-0.062)	0.003*
	Female	Reference (1)	
Professional education	Diploma in nursing	9.891 (3.664-26.698)	<0.001
	Degree	Reference (1)	
Socioeconomic status^#^	Upper middle class	0.627 (0.269-1.458)	0.278
	Lower middle class	Reference (1)	
Working area	COVID-19	0.146 (0.034-0.622)	0.009*
	Non-COVID-19	Reference (1)	
COVID-19 training	No	6.272 (2.751-14.298)	<0.001
	Yes	Reference (1)	
Contact with COVID-19-positive cases^$^	No	2.299 (1.222-4.713)	0.023*
	Yes	Reference (1)	
Quarantine days	≤14	3.175 (1.549-6.508)	0.002*
	>15	Reference (1)	
Family members in the medical profession	No	2.116 (1.000-4.479)	0.050
	Yes	Reference (1)	
Professional experience (years)	≤5	0.361 (0.175-0.746)	0.006*
	>6	Reference (1)	

All the variables that found a significant association in bivariate logistic regression were entered into a multivariable logistic regression to see the combined effect of nurses' characteristics on anxiety levels during the COVID-19 pandemic. The findings revealed that nurses aged <30 years and those who had diploma education in nursing felt more anxious as compared to nurses aged more than 30 years and had completed degree qualification in nursing (OR: 0.081, 95% CI: 0.015-0.424, p=0.003; OR: 22.247, 95% CI: 6.121-80.854, p<0.001, respectively). Nurses who had not attended training on COVID-19 reported seven times higher levels of anxiety than those who had completed training on different aspects of dealing with patients with COVID-19 (OR: 7.378, 95% CI: 1.980-27.497, p=0.003) (Table 5).

**Table 5:** Multivariable regression analysis to find the factors associated with anxiety levels among nurses (n=368) *Significant p-value at <0.05; ^$^lab-confirmed COVID-19 cases; ^ǂ^socioeconomic status and working areas are excluded from analysis considering negligible values in those sub-categories COVID-19: coronavirus disease 2019; OR: odds ratio; CI: confidence interval

Variables^ǂ^	Categories	OR (95% CI)	P-value
Age (years)	≤30	0.080 (0.015-0.424)	0.003*
	>30	Reference (1)	
Gender	Male	0.331 (0.058-1.897)	0.215
	Female	Reference (1)	
Professional education	Diploma in nursing	22.247 (6.121-80.854)	<0.001
	Degree	Reference (1)	
COVID-19 training	No	7.378 (1.980-27.497)	0.003*
	Yes	Reference (1)	
Contact with COVID-19-positive cases^$^	No	0.081 (0.006-1.143)	0.063
	Yes	Reference (1)	
Quarantine days	≤14	7.772 (0.612-97.398)	0.144
	>15	Reference (1)	
Family members in the medical profession	No	3.561 (0.982-12.911)	0.053
	Yes	Reference (1)	
Professional experience (years)	≤5	3.120 (0.733-13.272)	0.123
	>6	Reference (1)	

## Discussion

This study aimed to determine the factors associated with self-efficacy and anxiety levels among nurses working at a tertiary care hospital during the current COVID-19 pandemic. The mean score for self-efficacy in the present study is higher (32.19 ±4.53) than that in the Chinese (26.87 ±5.86) study [1] conducted during an early outbreak and another one performed in Taiwan (27.60 ±6.17) before the COVID-19 outbreak [23]. The positive change in self-efficacy may be attributed to the increased level of adaptation to the pandemic that had occurred over the course of a year, which had been fostered by an attitude to accept the new normal as well as acquiring more information on the pandemic. However, the difference in populations and settings might be another reason for this variation in the findings. The increasing level of self-efficacy in nurses indicates fewer mental health problems among them and a growing sense of better disaster preparedness [24]. Having higher levels of self-efficacy enables an individual to perform at a higher level by motivating them to get rid of the problems [25]. An individual with higher self-efficacy can better prepare and make a more effective contribution to fighting against a crisis situation such as a healthcare pandemic [26].

We found that socioeconomic status, training on COVID-19, living area, having family members tested positive for COVID-19, and the length of the quarantine period were significant contributors to nurses' levels of self-efficacy. Nurses in the high-income group had significantly higher levels of self-efficacy. It might be postulated that maintaining higher economic standards or getting a higher salary directly impacts the sense of self-efficacy. Moreover, a higher salary might be considered as deserved recognition of their challenging work during a pandemic [23]. Thus, the salary issue of nurses should be addressed by the nursing leaders, which may negatively impact self-efficacy, substantially decrease job satisfaction, and lead to high turnover rates [23,27]. We recommend that future studies examine the impact of self-efficacy on different organizational attributes and parameters such as job satisfaction, turnover, and actual performance.

The prevalence of anxiety was 9.2% among nurses in this study. These findings paint a more favorable and rosier picture compared to earlier studies conducted during the early periods of the outbreak, which have reported a higher prevalence of anxiety (40.8%) among Chinese nurses [28]. Likewise, the findings on psychological distress were surprisingly high (>70%) in another work conducted during the initial stage of the outbreak [12]. This high-risk situation related to psychological distress was reported among HCWs during the SARS outbreak too [29]. Likewise, anxiety was reported to be higher (40.8%) among nurses working in Singapore [30] and India [7] (48.54%) during the pandemic outbreak. According to another study, nurses of a designated hospital reported substantial psychological stress as well as symptoms of fear, anxiety, and compulsion [31].

It has been reported that HCWs reported substantially higher levels of psychological distress at the beginning of the pandemic compared to the general population [32], and most of them returned to states close to normalcy after a few months into the pandemic [33]. This could be attributed to the understandable psychological response of relief due to the passing of the pandemic's peak as well as hopes and expectations about returning to a normal life after the pandemic. Still, the authors advise that these findings be used cautiously. Of note, nurses reported more serious psychological problems than other HCWs [34], and this psychological distress might persist for a longer period of time and may worsen the current mental health crisis, by leading to conditions such as post-traumatic stress disorders [35,36].

Nurses' age, professional degree, and training related to caring for patients with COVID-19 were found to be significant predictors of anxiety among them. Nurses in the younger age group experienced less stress than older nurses. These findings are in concurrence with another study that reported a high prevalence of anxiety that coincided with increasing age in nurses. Likewise, the higher levels of stress in females in the present work are in line with another previous work [37]. A similar finding of higher levels of COVID-19-related anxiety in female nurses was reported in another study as well [38]. Further, nurses with higher professional education and those who have undergone training to handle the COVID-19 pandemic reported lower levels of stress. These findings align with an earlier study conducted during the initial outbreak of the pandemic in China, which reported lower rates of burnout and fear in nurses who have completed bachelor or higher degrees and undergone prior training related to caring for similar patients with viral infections [1]. Likewise, nurses who felt confident about caring for patients with COVID-19 reported lower levels of burnout and fear of handling pandemics such as the ongoing one [1]. Higher confidence in caring for the patient indicates prior preparedness gained by undergoing training related to COVID-19 or other similar viral infections in nurses, and in light of this, we recommend the implementation of more programs of similar nature that would provide appropriate training to other HCWs on different aspects of caring for patients with COVID-19.

Limitations

This study has some limitations. Primarily, the survey's cross-sectional nature did not equip us to determine the casualty or changes in stress and self-efficacy levels over time. Secondly, the study was conducted in a single institution, even though it was one of the region's largest centers; consequently, the findings' generalizability might be limited to this particular geographical region. Thirdly, the data on the impact of different COVID-19-related variables on anxiety and self-efficacy are scarce, and future research is required to rectify this, which would address all such variables. And finally, all questionnaires in this study were self-reported in nature and this may have led to reporting bias. Future research should preferably adopt a longitudinal approach to determine nurses' anxiety and self-efficacy levels during the ongoing pandemic.

## Conclusions

Based on our findings, a substantial number of nurses are still experiencing considerable levels of anxiety, even though many of them have acquired sufficient levels of self-efficacy after having worked through this pandemic for over a year. Many personal and professional characteristics of nurses have contributed to their levels of anxiety and self-efficacy. Hospital administrations should invest substantially more in terms of resources and programs for training the nursing staff on different aspects of caring for patients with COVID-19 to improve their levels of self-efficacy and anxiety while they are weathering this ongoing global pandemic.

## References

[1] Hu D, Kong Y, Li W (2020). Frontline nurses' burnout, anxiety, depression, and fear statuses and their associated factors during the COVID-19 outbreak in Wuhan, China: A large-scale cross-sectional study. EClinicalMedicine.

[2] (2021). World Health Organization: coronavirus disease (COVID-19) Situation Dashboard. https://covid19.who.int/.

[3] Kumar R, Singh V, Mohanty A, Bahurupi Y, Gupta PK (2020). Corona health-care warriors in India: knowledge, attitude, and practices during COVID-19 outbreak. J Edu Health Promot.

[4] Said NB, Chiang VCL (2020). The knowledge, skill competencies, and psychological preparedness of nurses for disasters: a systematic review. Int Emerg Nurs.

[5] Vijayaraghavan BKT, Myatra SN, Mathew M (2020). Challenges in the delivery of critical care in India during the COVID-19 pandemic. J Intensive Care Soc.

[6] Sribala Vadlapatla (2021). Hyderabad: 50 Niloufer Hospital staff quarantined. https://timesofindia.indiatimes.com/city/hyderabad/50-niloufer-staff-quarantined/articleshow/75240281.cms.

[7] Raj R, Koyalada S, Kumar A, Kumari S, Pani P, Nishant Nishant, Singh KK (2020). Psychological impact of the COVID-19 pandemic on healthcare workers in India: an observational study. J Family Med Prim Care.

[8] Zhu Z, Xu S, Wang H (2020). COVID-19 in Wuhan: sociodemographic characteristics and hospital support measures associated with the immediate psychological impact on healthcare workers. EClinicalMedicine.

[9] Simonetti V, Durante A, Ambrosca R (2021). Anxiety, sleep disorders and self-efficacy among nurses during COVID-19 pandemic: a large cross-sectional study. J Clin Nurs.

[10] Nacoti M, Ciocca A, Giupponi A (2020). At the epicenter of the covid-19 pandemic and humanitarian crises in Italy: changing perspectives on preparation and mitigation. NEJM Catal Innov Care Deliv.

[11] Kang L, Li Y, Hu S (2020). The mental health of medical workers in Wuhan, China dealing with the 2019 novel coronavirus. Lancet Psychiatry.

[12] Lai J, Ma S, Wang Y (2020). Factors associated with mental health outcomes among health care workers exposed to coronavirus disease 2019. JAMA Netw Open.

[13] Bansal P, Bingemann TA, Greenhawt M (2020). Clinician wellness during the COVID-19 pandemic: extraordinary times and unusual challenges for the allergist/immunologist. J Allergy Clin Immunol Pract.

[14] Farber EW, Schwartz JA, Schaper PE, Moonen DJ, McDaniel JS (2000). Resilience factors associated with adaptation to HIV disease. Psychosomatics.

[15] Yao Y, Zhao S, Gao X (2018). General self-efficacy modifies the effect of stress on burnout in nurses with different personality types. BMC Health Serv Res.

[16] Schwarzer R, Hallum S (2008). Perceived teacher self-efficacy as a predictor of job stress and burnout: mediation analyses. Appl Psychol.

[17] Fernandez R, Lord H, Halcomb E, Moxham L, Middleton R, Alananzeh I, Ellwood L (2020). Implications for COVID-19: a systematic review of nurses' experiences of working in acute care hospital settings during a respiratory pandemic. Int J Nurs Stud.

[18] Spitzer RL, Kroenke K, Williams JB, Löwe B (2006). A brief measure for assessing generalized anxiety disorder: the GAD-7. Arch Intern Med.

[19] Schwarzer R, Jerusalem M (1995). Generalized self-efficacy scale. Measures in Health Psychology: A User's Portfolio. Causal and Control Beliefs.

[20] Nilsson MH, Hagell P, Iwarsson S (2015). Psychometric properties of the General Self-Efficacy Scale in Parkinson's disease. Acta Neurol Scand.

[21] He Q, Ren J, Wang G, Zhang J, Xiang J, He D (2021). Psychological effects of the COVID-19 outbreak on nurses working in tertiary women's and children's hospitals from Sichuan, China: A cross-sectional study. Int J Disaster Risk Reduct.

[22] (2021). IBM Corp: IBM SPSS Statistics for Windows: Version 23.0. New York, NY, US. https://www.ibm.com/support/pages/how-cite-ibm-spss-statistics-or-earlier-versions-spss.

[23] Hu SH, Yu YM, Chang WY, Lin YK (2018). Social support and factors associated with self-efficacy among acute-care nurse practitioners. J Clin Nurs.

[24] Kılıç N, Şimşek N (2019). The effects of psychological first aid training on disaster preparedness perception and self-efficacy. Nurse Educ Today.

[25] Delgado C, Upton D, Ranse K, Furness T, Foster K (2017). Nurses' resilience and the emotional labour of nursing work: an integrative review of empirical literature. Int J Nurs Stud.

[26] Sumer N, Karanci AN, Berument SK, Gunes H (2005). Personal resources, coping self-efficacy, and quake exposure as predictors of psychological distress following the 1999 earthquake in Turkey. J Trauma Stress.

[27] Kim S, Kim S (2001). Self-efficacy and its impact on pay satisfaction, pay-level satisfaction, and benefits satisfaction. Seoul J Business.

[28] Xiong H, Yi S, Lin Y (2020). The psychological status and self-efficacy of nurses during COVID-19 outbreak: a cross-sectional survey. Inquiry.

[29] Chua SE, Cheung V, Cheung C (2004). Psychological effects of the SARS outbreak in Hong Kong on high-risk health care workers. Can J Psychiatry.

[30] Tan BYQ, Chew NWS, Lee GKH (2020). Psychological impact of the COVID-19 pandemic on health care workers in Singapore. Ann Intern Med.

[31] Zhu Huarong, Chen Fei, Tao Li (2021). Research on the psychological stress status and influencing factors of clinical nurses in designated hospitals for medical treatment during the prevention and control of new coronavirus pneumonia. Mod Clin Nurs.

[32] Sasaki N, Kuroda R, Tsuno K, Kawakami N (2020). The deterioration of mental health among healthcare workers during the COVID-19 outbreak: a population-based cohort study of workers in Japan. Scand J Work Environ Health.

[33] Wang C, Pan R, Wan X (2020). A longitudinal study on the mental health of general population during the COVID-19 epidemic in China. Brain Behav Immun.

[34] Nickell LA, Crighton EJ, Tracy CS (2004). Psychosocial effects of SARS on hospital staff: survey of a large tertiary care institution. CMAJ.

[35] Lung FW, Lu YC, Chang YY, Shu BC (2009). Mental symptoms in different health professionals during the SARS attack: a follow-up study. Psychiatr Q.

[36] Lancee WJ, Maunder RG, Goldbloom DS (2008). Prevalence of psychiatric disorders among Toronto hospital workers one to two years after the SARS outbreak. Psychiatr Serv.

[37] Tsaras K, Papathanasiou IV, Vus V, Panagiotopoulou A, Katsou MA, Kelesi M, Fradelos EC (2018). Predicting factors of depression and anxiety in mental health nurses: a quantitative cross-sectional study. Med Arch.

[38] Bidzan M, Bidzan-Bluma I, Szulman-Wardal A, Stueck M, Bidzan M (2020). Does self-efficacy and emotional control protect hospital staff from COVID-19 anxiety and PTSD symptoms? psychological functioning of hospital staff after the announcement of COVID-19 coronavirus pandemic. Front Psychol.

